# No Difference in Sleep Desaturations Severity between Patients with Wake-Up and Non-Wake-Up Stroke: A PRESS Study Results

**DOI:** 10.3390/life13020517

**Published:** 2023-02-13

**Authors:** Katarína Klobučníková, Branislav Kollár, Matúš Jurík, Katarína Valovičová, Miroslava Hardoňová, Michal Poddaný, Miroslav Tedla, Michal Riant, Pavel Klail, Peter Turčáni, Pavel Šiarnik

**Affiliations:** 1 1st Department of Neurology, Faculty of Medicine, Comenius University, 814 69 Bratislava, Slovakia; 2 Department of Neurology, General Hospital, 031 23 Liptovsky Mikulas, Slovakia; 3 Department of ENT and HNS, Faculty of Medicine, University Hospital Bratislava, Comenius University, 814 69 Bratislava, Slovakia; 4 Institute of Cancer and Genomic Sciences, University of Birmingham, Birmingham B15 2T8, UK; 5 Department of Otorhinolaryngology, University Hospital, Faculty of Medicine in Pilsen, Charles University, 100 00 Prague, Czech Republic

**Keywords:** ischemic stroke, wake-up stroke, pulse oximetry, sleep-disordered breathing

## Abstract

Background: Wake-up stroke (WUS) is a certain type of ischemic stroke in which a patient wakes up with a new neurological deficit due to cerebral ischemia. Sleep-disordered breathing is an independent risk factor for stroke, but the role of nocturnal oxygen desaturation in the pathophysiology of WUS is still insufficiently explored. According to several studies, patients with WUS have a significantly more severe sleep apnea syndrome and lower mean blood oxygen saturation. This study aimed to assess the severity of nocturnal desaturations in acute WUS and non-WUS patients using nocturnal pulse oximetry. Material and Methods: The cohort of 225 consecutive patients with neuroimaging-verified acute cerebral ischemia was prospectively enrolled. For further analyses, 213 subjects with known WUS/non-WUS status were selected (111 males and 102 females, average age 70.4 ±12.9, median baseline NIHSS = 5, median baseline mRS = 3). Patients were divided into the WUS group (n = 45) and the non-WUS group (n = 168). Overnight pulse oximetry was performed within 7 days of the stroke onset and data of both of the studied groups were compared. Results: We found oxygen desaturation index (ODI) in the WUS group was 14.5 vs. 16.6 (*p* = 0.728) in the non-WUS group, basal O2 saturation was 92.2% vs. 92.5% (*p* = 0.475), average low O2 saturation was 90.3% vs. 89.6% (*p* = 0.375), minimal O2 saturation was 79.5% vs. 80.6% (*p* = 0.563), and time with O2 saturation <90% (T90) was 4.4% vs. 4.7% (*p* = 0.729). Conclusions: In the studied sample, monitored respiratory parameters including ODI, basal O2 saturation, average low O2 saturation, minimal O2 saturation, and T90 did not significantly differ between groups of WUS and non-WUS patients.

## 1. Introduction

Wake-up stroke (WUS) is a certain type of ischemic stroke in which a patient wakes up with a new neurological deficit due to cerebral ischemia. Up to 14–25% of patients with acute stroke suffer WUS [1,2]. As the exact time of stroke onset is unknown, these patients are at risk of losing the potential benefit of revascularization therapy [3]. Sleep-disordered breathing (SDB) is an independent risk factor for stroke, but the role of nocturnal oxygen desaturation in the pathophysiology of WUS is still insufficiently explored [4]. According to several studies, patients with WUS have significantly higher severity of sleep apnea and lower mean blood oxygen saturation [5,6,7]. A recent meta-analysis of 13 studies’ results showed that sleep apnea syndrome (SAS) is significantly higher in WUS patients, with significantly higher apnea-hypopnea index (AHI; 95% confidence interval: 1.38–14.11; *p* = 0.017) and oxygen desaturation index (ODI; 95% confidence interval: 0.261–7.438; *p* = 0.035) [8]. Increased risk of stroke in these patients may be mediated by hemodynamic changes due to sleep apnea, oxidative stress, endothelial dysfunction, sympathetic overactivity, metabolic dysregulation, accelerated hypertension, paradoxical embolism, or arrhythmogenesis [9,10]. Polysomnography (PSG) is a gold standard diagnostic tool for the complex estimation of SDB. However, this method is suitable only for cooperating patients. The clear advantage of PSG is the information on the relationship between the occurrence of apneic episodes and the stages of sleep, including the hypnogram, but limits of PSG include that it requires educated medical staff and monitoring devices, and causes patients discomfort. Several studies proved overnight pulse oximetry is a simple method to evaluate nocturnal desaturations in patients with stroke [11,12,13]. This method can be used as a sensitive screening tool in this population [14,15,16]. This study aimed to explore and compare the severity of nocturnal desaturations in a “real world” population of patients with acute WUS and non-WUS.

## 2. Materials and Methods

### 2.1. Materials

**Figure 1:**
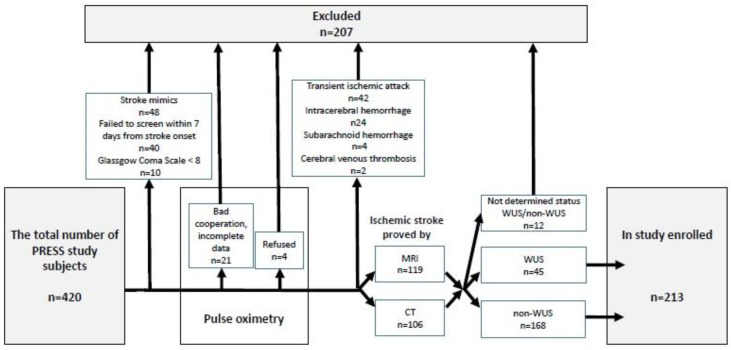
Flowchart.

The study subjects were enrolled from the population of stroke subjects who were recruited within the PRESS study (pulse oximetric routine examination of sleep apnea in acute stroke), as previously described [14] (see flowchart in Figure 1).

We prospectively enrolled patients hospitalized in the 1st Department of Neurology, Comenius University Bratislava. The diagnosis of stroke was confirmed clinically, and computed tomography (CT) or magnetic resonance imaging (MRI) was used to confirm the site of the ischemic lesion. Only the subjects with neuroimaging-verified acute brain infarction were enrolled. WUS was defined as the occurrence of new stroke symptoms detected upon waking up from sleep and non-WUS status was set when symptoms were detected during the awake state; for demographic details see Table 1. Baseline stroke severity was measured by the National Institutes of Health Stroke Scale (NIHSS) and modified Rankin Scale (mRS) [17,18].

Inclusion criteria: patients from PRESS study, clinical diagnosis of acute stroke confirmed by CT or MRI, age over 18.

Exclusion criteria: not determined WUS/non-WUS status, diagnosis of acute stroke not-confirmed by CT or MRI, other than ischemic etiology of stroke, stroke mimics, stroke onset > 7 days before pulse oximetry, impairment of consciousness with the Glasgow coma scale < 8 on admission, bad cooperation (including severe disability, agitated confusion, acute cardiac/respiratory comorbidity, acute exacerbation of chronic cardiac/respiratory comorbidity), incomplete clinical data (impossibility to record at least 4 h of continuous pulse oximetric monitoring), refusal to participate.

### 2.2. Methods

Single-night pulse oximetry monitoring was performed as previously described [14]. Briefly, using the WristOx2 device (model 3150, Nonin Medical, Plymouth, MA, USA) nocturnal monitoring was performed from 10 p.m. to 6 a.m.

The following variables were recorded and analyzed:

*Desaturation* was defined as an event with a drop of oxygen level >3% and with a duration >10 s.

*Oxygen desaturation index (ODI)* was defined as the average number of desaturations during 1 h of recording.

*Basal O2 saturation* (steady-state O2 saturation) was defined as an average of the O2 saturation readings that were not included in any desaturation event.

*Average low O2 saturation* was defined as the average of the lowest O2 saturations seen over all respiratory events.

*Average event duration* was defined as the average duration of desaturation and expressed as an average time in seconds per event.

*Minimal O2 saturation* was defined as the lowest single O2 saturation seen in the recording.

*Time with O2 saturation <90*% (T90) was defined as the proportion of time period with O2 saturation <90% from the total duration of the recording.

### 2.3. Statistics

Continuous variables were expressed as means ± standard deviation or median, interquartile range (IQR), minimal and maximal values. Categorical variables were expressed as numbers and proportions (%). To compare variables in the WUS vs. non-WUS population, the Student *t* test, Mann–Whitney test, and χ2 test were used for particular variables. SPSS version 21 (SPSS Inc., Chicago, USA) was used for statistical analyses. *p* values < 0.05 were considered statistically significant.

### 2.4. Ethics

The study was approved by the Ethics Committee of the Old Town Hospital, University Hospital Bratislava on 15th April 2019. All the patients or their next of kin signed the informed consent before being recruited.

## 3. Results

In our study, out of 225 patients with neuroimaging verified acute cerebral ischemia, 213 subjects had known WUS/non-WUS status (111 males and 102 females, average age 70.4 ±12.9, median baseline NIHSS = 5, median baseline mRS = 3). This sample was selected for further analyses. We compared the data of the 45 patients WUS group and the 168 patients in the non-WUS group. Between WUS and non-WUS groups, no significant gender and age differences were found (*p* = 0.443 and *p* = 0.611).

Stroke severity was measured by the National Institutes of Health Stroke Scale and modified Rankin Scale. Both methods showed insignificant differences in stroke severity in compared groups (*p* = 0.743, *p* = 0.763, consecutively).

Oximetry-related indices commonly used in SAS examination were also without disparity. Oxygen desaturation index was 14.5 vs. 16.6 (*p* = 0.728), basal O2 saturation 92.2% vs. 92.5% (*p* = 0.475), average low O2 saturation 90.3% vs. 89.6 % (*p* = 0.375), minimal O2 saturation 79.5% vs. 80.6% (*p* = 0.563) and T90 was 4.4% vs. 4.7% (*p* = 0.729). See Table 2 for more details.

## 4. Discussion

From the total number of 420 patients from PRESS study [14], only 213 subjects fulfilled the inclusion criteria (102 females, 111 males). The studied sample included in the work represented the “real world” population (female age median 75.5; male 68.0 years) and the respondents were divided into two well comparable groups: WUS group with 22 females (48.9%), age median 73.5 years and 23 males (51.1%), age median 70.0 years; versus NWUS-group with 80 females (47.6%), age median 76.5 years and 88 males (52.4%), age median 67.5 years.

A “gold standard” diagnostic tool for sleep apnea, polysomnography, is used in the majority of the studies [19,20,21], although other simpler sleep monitoring tools of home polygraphy (PG) are also used [22,23]. The use of questionnaires (Berlin questionnaire, STOP-BANG, Epworth Sleepiness Scale) in sleep apnea assessment in stroke subjects is limited by their low sensitivity and specificity [24,25]. PSG, like the most complex investigation, provides information on all important markers of SAS including a hypnogram but it is connected with patient hospitalization-associated stress and is equipment-demanding (educated staff as well as expensive devices). PG gives less information, but it is performed on patients in their home environment. For these reasons, research into less demanding options for sleep monitoring examinations is ongoing [26,27]. Except for the fact that PSG is a technically demanding and costly examination with limited availability, the feasibility of PSG in acute stroke patients represents one of the most challenging issues even in departments with close cooperation between the stroke unit and sleep laboratory [16,19]. All these issues limit routine polysomnographic assessment in a “real world” population of patients with acute stroke. Nocturnal pulse oximetry in stroke subjects represents an alternative diagnostic method with excellent adherence and good sensitivity (90.5%) [14,28,29]. However, lower specificity (75%) may limit the interpretation of the findings described [14].

The high prevalence of sleep apnea in stroke subjects is well-known, and was described in 2005 by Bassetti and Artzt et al. [4,30]. According to a meta-analysis from 2019 by Seiler and his team, it is present in 71% [31]. A large recent work from 2022 by Lu and Liu proved the prevalence of sleep apnea might be even higher in the WUS population. Their meta-analysis included 13 studies and showed that SAS-prevalence is significantly higher in WUS patients [8], with a significantly higher severity of sleep apnea according to AHI and ODI (*p = 0.017*, and *p = 0.035*). This topic was researched by Barreto et al., Haula et al., and Schütz et al. [32,33,34] and the results of cited studies suggested that preexisting sleep apnea is associated with the occurrence of WUS or even with worse short-term outcomes measured in mRS [33]. Kim et al. in 2018, using the Berlin Questionnaire [10], found that preexisting witnessed or self-recognized sleep apnea was significantly more frequent in WUS when compared to the non-WUS population (28.3% vs. 16.6%, *p* = 0.036). In the same study, the presence of preexisting witnessed or self-recognized sleep apnea was the only risk factor for WUS (adjusted odds ratio = 2.055, 95% confidence interval = 1.035–4.083, *p =* 0.040) [10]. Their conclusion is not in agreement with our study. We did not prove the higher severity of sleep apnea in WUS. In a comparison of WUS and non-WUS groups, we found median ODI 14.5 vs. 16.6 (*p* = 0.728) and T90 4.4% vs. 4.7% (*p* = 0.729). For details, see Table 2. Similarly to our study, also other studies using portable devices failed to find an association between sleep apnea and WUS [32,35]. In our case, we suppose that the population enrolled in the current study may better reflect the “real world” acute stroke population than populations in polysomnographic studies.

Early sleep apnea assessment (nocturnal pulse oximetry evaluation within seven days from the stroke onset) was performed in the current study. It is necessary to admit that Slonková et al. previously described spontaneous improvement of sleep apnea in stroke patients [36], and these findings were supported also by the results of a recent study by Šiarnik et al. [16]. It remains unknown if there is any significant difference in spontaneous OSA improvement between WUS and non-WUS subjects and future research in this field is warranted. However, later sleep apnea assessment could influence the results of our current study. The optimal timing of sleep apnea assessment post-stroke also needs to be elucidated by future prospective studies, because it can influence further therapeutic approaches and treatment adherence in this high-risk population [16].

We are aware of the fact that the low specificity of the pulse oximetry examination in sleep apnea assessment represents one of the main limitations of the current study. PG seems to be a promising alternative diagnostic tool in stroke subjects [22,23]. We have to admit that the PRESS study [14] was not initially designed to search for predictors of nocturnal desaturations. Multiple variables, including stroke comorbidities and therapy, could play a more important role in nocturnal oxygen desaturation than WUS/non-WUS status itself. In the retrospective search of the patients’ records, we found none of the 213 analyzed subjects was treated with positive airway pressure therapy pre-stroke. Similarly none of these patients was treated by oxygen therapy during the diagnostic night. However, multiple other sleep apnea-related conditions beyond these should be considered in future prospective studies, including respiratory tract disorders and cardiac disorders.

## 5. Conclusions

In our studied sample, monitored respiratory parameters including ODI, basal O2 saturation, average low O2 saturation, minimal O2 saturation, and T90 did not significantly differ between groups of WUS and non-WUS patients.

## Figures and Tables

**Table 1:** Demographic data of the studied sample.

Group	Female				Male				Total				
	n	%	Age		n	%	Age		n	%	Age		
			Mean	Median			Mean	Median			Mean	Median	*p*-Level
WUS	22	21.6	71.8	73.5	23	20.7	67.3	70.0	45	21.1	69.5	72.0	0.611
non-WUS	80	78.4	73.9	76.5	88	79.3	67.6	67.5	168	78.9	70.6	72.0	
Total	102	100	73.4	75.5	111	100	67.6	68.0	213	100	70.4	72	

WUS: Wake-up stroke.

**Table 2:** Comparison of monitored variables in WUS vs. non-WUS stroke groups.

Group				WUS					Non-WUS			*p*
				N = 45					N = 168			---
Gender	Male (n)	23		51.1%			88		52.4%			0.443
	Female (n)	22		48.9%			80		47.6%			
		Mean	SD	Median	IQR	Range	Mean	SD	Median	IQR	Range	
Age (years)		69.5	12.6	72.0	14.5	28.0–94.0	70.6	13.0	72.0	18.0	23.0–97.0	0.611
NIHSS on admission		6.8	5.3	5.0	6.0	1.0–20.0	6.8	5.7	5.0	8.0	1.0–33.0	0.743
mRS on admission		3.1	1.5	3	2.5	1.0–5.0	3.1	1.5	3.0	3.0	1.0–5.0	0.763
ODI (n/h)		21.6	17.8	14.5	19.6	2.8–69.4	23.2	19.1	16.6	24.4	1.1–74.9	0.728
	ODI ≥ 5 (n)	41		91.1%			148		88.1%			0.570
	ODI ≥ 15 (n)	25		55.5%			77		45.8%			0.246
	ODI ≥ 30 (n)	10		22.2%			46		27.4%			0.485
Average event duration (s)		38.9	15.0	35.6	16.9	21.1–87.1	39.0	12.4	37.0	15.5	20.6–98.4	0.985
Basal O2 saturation (%)		92.2	2.2	92.6	2.3	83.3–95.6	92.5	2.0	92.9	2.5	86.4–97.3	0.475
Average low O2 saturation (%)		90.3	2.3	90.7	2.7	81.6–93.8	89.6	4.9	90.5	3.1	34.9–95.1	0.375
Minimal O2 saturation (%)		79.5	9.6	82.0	10.5	50.0–90.0	80.6	9.2	83.0	9.0	39.0–93.0	0.563
T 90 (%)		13.4	19.4	4.4	21.6	0–95.8	15.7	22.6	4.7	21.6	0–95.8	0.729

IQR: interquartile range, mRS: modified Rankin Scale, NIHSS: National Institutes of Health Stroke Scale, ODI: oxygen desaturation index, SD: standard deviation, T90: time with O2 saturation < 90%, WUS: Wake-up stroke.

## Data Availability

The data presented in this study are available on request from the corresponding author.

## Abbreviations

AHI	apnea-hypopnea index
CT	computed tomography
MRI	magnetic resonance imaging
mRS	modified Rankin Scale
NIHSS	National Institutes of Health Stroke Scale
ODI	oxygen desaturation index
OSA	obstructive sleep apnea
PG	polygraphy
PSG	polysomnography
SAS	sleep apnea syndrome
SDB	sleep-disordered breathing
T90	time with O_2_ saturation < 90%
WUS	wake-up stroke
